# Micro-XCT analysis of anatomical features and dimensions of the incisive canal: implications for dental implant treatment in the anterior maxilla

**DOI:** 10.1186/s12903-024-05046-3

**Published:** 2024-10-18

**Authors:** Vladimir S. Todorovic, Mia-Michaela Beetge, Judy Kleyn, Jakobus Hoffman, Andre W. van Zyl

**Affiliations:** 1 School of Dental Medicine, University of Belgrade, Belgrade, 11000 Serbia; 2 Department of Periodontics and Oral Medicine, Faculty of Health Sciences, University of Pretoria, Pretoria, South Africa; 3 Head Clinical Unit, Department of Periodontics and Oral Medicine, Faculty of Health Sciences, University of Pretoria, Pretoria, South Africa; 4 Department of Statistics, Faculty of Natural and Agricultural Sciences, University of Pretoria, Pretoria, South Africa; 5 Necsa (The South African Nuclear Energy Corporation), Pretoria, South Africa; 6 Private Practice, Western Cape, South Africa

**Keywords:** Micro-XCT, Dental implants, Maxillary bone, Anatomy, Maxillary incisive canal

## Abstract

**Background:**

This study used micro-focus X-ray Computed Tomography (micro-XCT) to examine the anatomical differences and dimensions of the maxillary incisive canal (MIC) in a South African population. The accurate imaging yielded dependable results that support earlier research and enhance anterior maxilla surgery planning. Furthermore, these anatomical features are compared between various racial and gender groupings in the study.

**Methods:**

Using a micro-XCT scanner, 108 human cadaver skulls from the Pretoria Bone Collection were scanned and included in the study. Advanced volume rendering software was employed for measuring the MIC length, diameter, shape, and the buccal bone wall measurements in relation to the MIC.

**Results:**

Significant anatomical variation in the size and shape of the MIC was identified in the population, with variations seen between racial and gender groups. The incisive foramen (ICO) mean diameter was 6.61 mm, and the MIC length varied from 4.96 to 20.10 mm. There were significant differences in the buccal alveolar bone height between different ethnic groups and gender. Regarding morphological patterns in coronal and sagittal views, single canals were more common in the black population while Y-shaped canals were more common in the white population. The study also introduced a new metric by measuring the mean distances between teeth #11 and #21 and the ICO (1.83 mm and 1.88 mm respectively).

**Conclusions:**

The complex anatomical differences of the MIC in a South African population were clarified. Clinicians should be aware of tooth sockets in near proximity to the MIC and perform accurate preoperative assessment using sophisticated 3-D imaging and preferable guided implant placement in the anterior maxilla.

## Introduction

Clinical anatomy represents the foundation for surgical interventions and forms the basis for diagnosis, treatment, and aftercare [1]. Anatomical differences between individuals are well-documented and can influence clinical outcomes. Failure to consider these variations may result in atypical symptom presentations, which can complicate clinical examinations and the interpretation of imaging results [2]. Intra- and post-operative complications, such as lack of dental implant osseointegration, nerve damage, and poor prosthodontic outcomes can all result from inadequate anatomical awareness [1, 3–6]. Therefore, it is crucial to have a comprehensive knowledge of clinical anatomy and any potential individual anatomical differences to execute safe and successful oral surgical procedures such as implant placement and application of local anaesthetic in the anterior maxilla [1, 7, 8].

The maxillary incisive canal (MIC), also called in the literature the nasopalatine canal (NC) or anterior palatine canal, has been described as a canal located in the middle of the palate, posterior to the roots of the central maxillary incisors [4, 7, 9, 10]. To define the terminology adequately and avoid any confusion, this study will employ the nomenclature established by Song and colleagues (2009): (1) the canal is referred to as the MIC (nasopalatine canal is another term used that is anatomically correct); (2) the inferior opening known as the incisive foramen (ICO) and; (3) the superior opening is referred to as either the nasal opening(s) (ICN) or the foramen of Stenson [11]. The ICO is typically found directly beneath the incisive papilla in the midline of the anterior palate [9]. The MIC is an important connection between the nasal and oral cavities, containing the nasopalatine nerve, descending branch of nasopalatine artery, fibrous connective tissue, and minor salivary glands [4, 7, 10]. The morphology and size of the MIC have been reported by a number of researchers [1, 4–6, 8–10, 12–16]. The MIC can have up to four canals in the midline, two to five ICN’s and one to three ICO’s [3, 5, 11, 17]. Sicher and colleagues (1962) reported up to six separate ICN’s [18]. When there are many foramina, it is thought that the neural and vascular components are also divided [6, 18]. In order to prevent harming the neurovascular systems in the MIC and subsequent osseointegration failure, dental implant placement should protect all neural canaliculi [3]. Bilateral canals with openings on either side of the palatal incisive papilla are rare and is more prevalent in other mammals, such baboons or dogs, and is linked to the Jacobson accessory vomeronasal organs, which have smell and taste receptors [19].

Previous studies have revealed differences in the MIC size and morphology, as well as the thickness of the anterior maxilla bone in relation to age, gender, edentulism, and ethnicity [5, 7–13, 20, 21]. In the dentulous maxilla, the typical MIC diameter is less than 6 mm; if this value is exceeded, conditions including canal cysts, trauma, and tooth loss may be present [22]. This contrasts with the findings of Mraiwa et al., who observed a diameter of up to 9.2 mm even in the absence of pathology [6]. Several studies revealed how gender and the presence of teeth affect anatomical features [6, 9, 11, 13, 17, 21]. It has been suggested that care should be taken with younger and female patients during surgical procedures due to root proximity to the MIC at the mid-root level of the maxillary central incisors [10, 12, 13, 23].

For evaluating the size and morphology of the MIC, various modalities have been employed and documented in the literature [5, 7–13, 20, 21]. Diagnostic imaging for the anterior maxilla has included two-dimensional (2-D) methods (intra-oral radiography and panoramic imaging) and cross-sectional or multiplanar-reformatted computed tomography (CT) [24]. The introduction of cone-beam computed tomography (CBCT) has opened up new diagnostic opportunities in dentistry [25]. Three-dimensional (3-D) imaging techniques allows a thorough anatomical examination within all three planes (axial, sagittal, and coronal) [7]. The MIC can be assessed using CT scans [6, 13, 17], micro-XCT images [11, 20], high resolution magnetic resonance imaging [26] and limited CBCT [9].

There is a data gap regarding African cohorts because previous studies on the anatomical features and size of the MIC have mostly focused on Western populations. To fill this gap, this study analysed the maxillary IC in a population of South Africans utilizing high-resolution micro-XCT imaging. Although a few studies have investigated age- and gender-related variations in MIC morphology, there aren’t many thorough analyses that compare racial and gender characteristics. This study investigated these factors to better understand MIC architecture for improved surgical results and dental implant planning in different groups.

### Clinical application

In the anterior maxilla, immediate implant placement is frequently chosen as a treatment option since it shortens recovery time and requires less surgical procedures [12, 27]. Despite its popularity, the protocol of immediate implant placement is still debatable, as it comes with a high risk of complications, especially when there is insufficient buccal and palatal alveolar bone [27, 28]. Implant failure might result when there is insufficient palatal bone with perforation into palatal soft tissues or MIC [23, 29]. A study by Alkanderi (2019) showed that approximately 8% of cases planned for immediate implant placement may perforate the maxillary IC [30]. It is well known that primary implant stability is one of the prerequisites for successful implant osseointegration [28, 31]. To achieve primary stability during immediate implant placement, between 3 and 5 mm of bone is needed beyond the apex and toward palatal direction to place a dental implant of at least 10 mm in length [31]. Todorovic et al. (2023); concluded that although the alveolar bone on the palatal aspect was thicker compared to the buccal side, the crestal bone can present with dimensions less than 1 mm [27]. In the severely atrophied maxilla, surgical procedures such as MIC grafting and nerve displacement have been described, however these procedures are not commonly employed [4, 14, 23, 32–35]. A single, cylindrical maxillary IC appears to be the most suitable anatomic variation for such procedures [14]. Despite the fact that the majority of studies show no altered sensation following MIC grafting, the literature lacks comprehensive information on the dangers and clinical repercussions of harming the canal and its neurovascular structures (14, 23, 32–35). When the MIC is violated during oral surgical procedures like central incisor apicoectomies, enucleation of nasopalatine duct cysts, LeFort 1 osteotomies, surgically assisted rapid palatal expansion, and dental implant placement, it can result in surgical complications such as sensory dysfunction or non-osseointegration of implants [6, 7, 23].

Only a few studies have used micro-XCT to investigate the micro-anatomy of the MIC [11, 20]. Kim and associates reported on the usefulness of micro-XCT in analysing the internal microstructure of bones [29]. Micro-XCT has an imaging resolution of 5 μm at the maximum, making it possible to evaluate complex structural properties [29]. In our study, micro-XCT was utilized to quantify the maxillary IC dimensions, assess its anatomical properties, and determine the buccal wall dimensions of the MIC. This is the first instance of micro-XCT being employed for this function that we are aware of.

## Materials and methods

### Study design

This retrospective, cross-sectional study analysed and included existing data from 108 human cadaver skulls to examine the anatomical features of the MIC. This descriptive study provides detailed measurements and descriptions, and correlational as it explores the relationship between the anatomical features and sociodemographic variables such as gender and race.

### Setting

The skeletal material was scanned at high resolution in the Micro-Focus X-ray Radiography and Tomography Facility (MIXRAD) of the South African Nuclear Energy Corporation, South Africa (NECSA) using a Nikon XTH 225 ST industrial Computed Tomography system (Nikon XTH 225 ST, Nikon Corporation, Japan).

### Participants

Skeletal material (skulls) from the Pretoria Bone Collection have been used in this study. The research collection has a proud history that started in August 1942 [36, 37]. The skulls that were scanned were accessioned into the collection between 1990. and 2012. Upon arrival at the Department of Anatomy, an accession number was given to the deceased, which was recorded in the cadaver registry. This number was linked to the individual’s personal details. The body was then embalmed and placed in storage for 1–2 years before dissection of the cadavers took place [36]. Following dissection, the cadavers were macerated and processed into skeletal elements [37]. The criterion for the skeletal remains to be included in the research collection, is that age, sex and population affinity of the individuals were known [36]. The skulls were then stored in acid-free cardboard boxes in temperature-controlled rooms to ensure their preservation and integrity.

The following were the inclusion criteria for this study: (a) Anatomically complete maxillary IC and surrounding maxillary bone, (b) presence of maxillary central incisors and (c) available demographic information. The exclusion criteria were (a) impacted teeth in the region of interest, (b) presence of a radiolucent, radiopaque, or mixed radiolucent-radiopaque lesion in the region of interest; (c) dental implants or bone grafts in the region of interest. Additionally, the patients’ edentulous status was noted and excluded when the central incisors were missing in this study, which may have an effect on the anatomical structure due to maxillary resorption.

Sociodemographic variables were categorized into two groups: gender (male/female) and population (Black/White).

### Measurements

For each skeletal element the best scanning parameters were selected according to the size and the density of the bone. Due to the specimen size in this study, a spatial resolution of less than 90 microns for the respective tomograms of the maxillae were achieved. This resulted in a much higher quality tomogram (3-D image) than CBCT from which more accurate quantitative analyses could be made [38]. Each of the 2-D digitized radiographs per specimen, taken at different angles, consisted of an array of 2048 × 2048-pixel elements (maximum for the current detector at the micro-XCT device) and each element with a 16-bit gray scale (65535 Gy levels). The reconstruction into 2-D slices (for each row of the 2048 × 2048-pixel array), was performed through Nikon CT-Pro 3-D software, a commercial tomography reconstruction package for micro-XCT, which created a single virtual 3-D volume file by reconstructing all the 2-D slices together with all the information of the sample.

The volume files were imported into advanced volume rendering software (VGStudio MAX 2.2, Volume Graphics GmbH, Germany) for the 3-D rendering, segmentation, and visualisation of the reconstructed volume data. The software offers quantitative analysis of the virtual volume and a menu of analytical functions is available, so that distances in 3-D space can be measured by integrating the information provided by the 3-D image together with the axial, sagittal and coronal views which show the additional xy, yz and xz slices, respectively. The specimen is defined through a density map of constituents of the sample as the virtual volume is being defined by 3D voxel elements, each with a different voxel value representing its density (up to 56535 Gy values), enabling the possibility of porosity quantification throughout the sample.

### Evaluation of images

The Frankfort horizontal plane was created on the 3-D model as a line approximating the base of the cranium, passing from the infraorbital ridge to the midline of the occiput, intersecting the superior margin of the external auditory meatus. This allowed for the cranium to be in the anatomic position where the base of the skull lies in the correct horizontal plane and where right and left sides are level. A repeatable reference plane allowed us to orientate all skulls in a similar manner from which measurements could be taken.

### Anatomical characteristics of the IC

Coronal and sagittal views were used to evaluate the anatomic characteristics of the MIC. In sagittal slices, the anatomic variants of the MIC were classified into four groups: [1] cylinder-shaped; [2] funnel-shape; [3] banana-shaped and [4] hourglass-shaped, in accordance with Mardinger and Mraiwa classifications [6, 13]. In coronal slices, the anatomic variants of the MIC were classified into three groups according to Bornstein’s proposed classification (2011): [1] a single canal; [2] two parallel canals and [3] variations of the Y-type canal with one oral opening (ICO) and two or more nasal openings (ICN) [9].

### Measurements to determine dimensions of the IC

The dimensions of the MIC were measured in millimetre, to the second decimal. Measurements were taken using all three anatomical planes (axial, sagittal and coronal). The following landmarks were selected for standardized measurements [1] the diameter of the foramina of Stenson (ICN) was measured at the level located on the axial plane where the ICN was at its largest (when two or more nasal openings were present, the diameters of all nasal openings were added together an a mean value was calculated); [2] diameter of the incisive foramen (ICO) was measured at the level located on the axial plane when the incisive foramen of the MIC was at its largest (when more than one oral opening was present, the diameters of all openings were added together, and the mean value was calculated); [3] MIC length (ICL) was defined as the distance from the ICO to the ICN. When two parallel canals or canals with a Y-shaped morphology were present, the length was calculated as a mean value of the different canal measurements.

### Measurements to determine dimensions of the buccal bone wall in relation to the IC

**Fig. 1:**
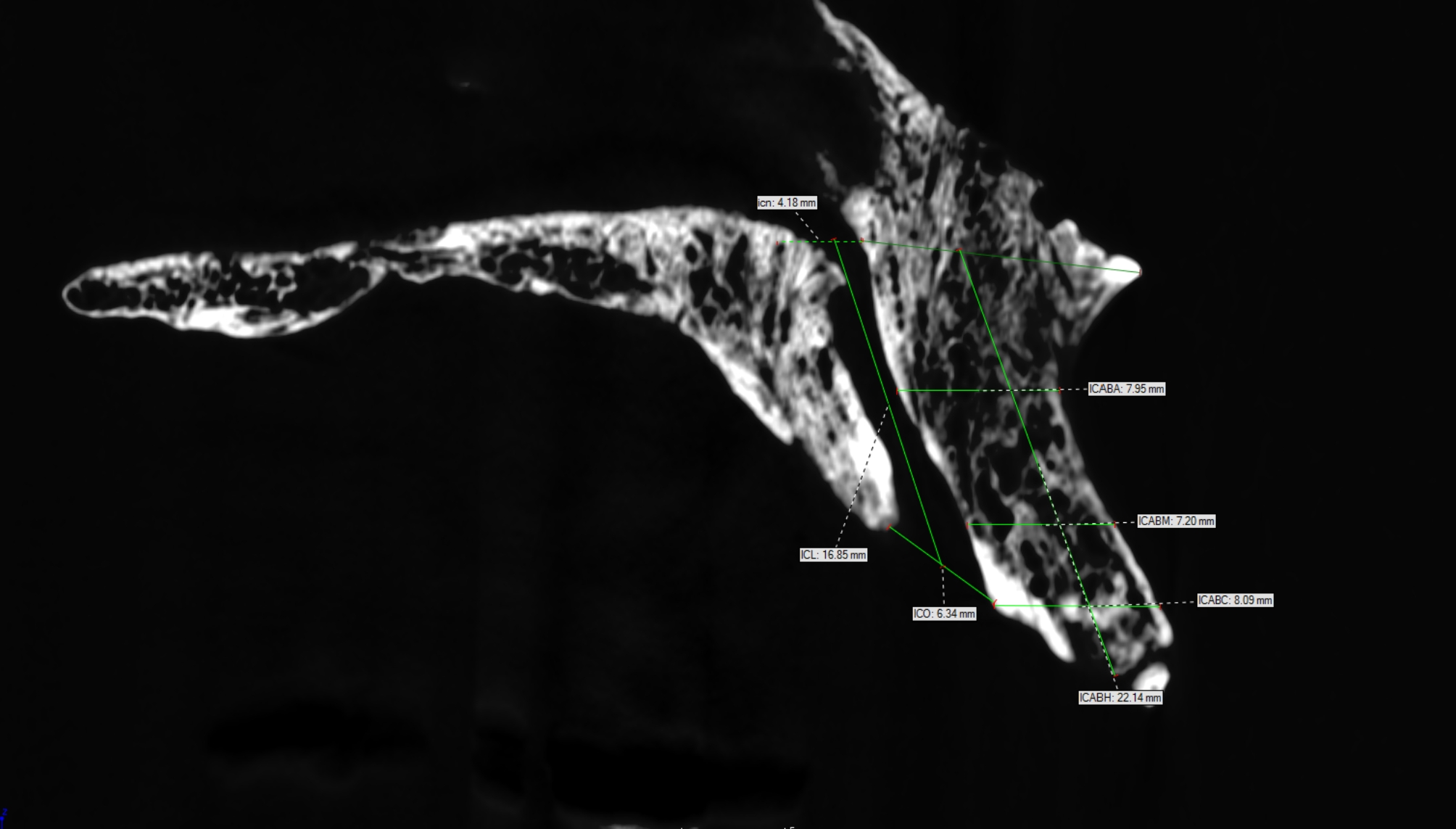
All measurement performed in sagittal view

[4] The most crestal measurement (ICABC) evaluated the distance from the buccal border of the ICO to the facial aspect of the buccal bone plate; [5] the second measurement was taken at the level opposite the palatal border of the ICO to the facial buccal bone wall (ICABM); [6] the most cranial (apical) reading (ICABA) evaluated the distance from the buccal border in the middle of the IC to the facial aspect of the buccal bone wall; [7] the height of the buccal alveolar bone (ICABH) was measured as follows: a line was drawn from the ICN to the most prominent point of the nasal spine. A second line was drawn perpendicular to this line and extended to the most coronal point of the alveolar crest. All measurement performed in sagittal view are displayed in Fig. 1.

**Fig. 2:**
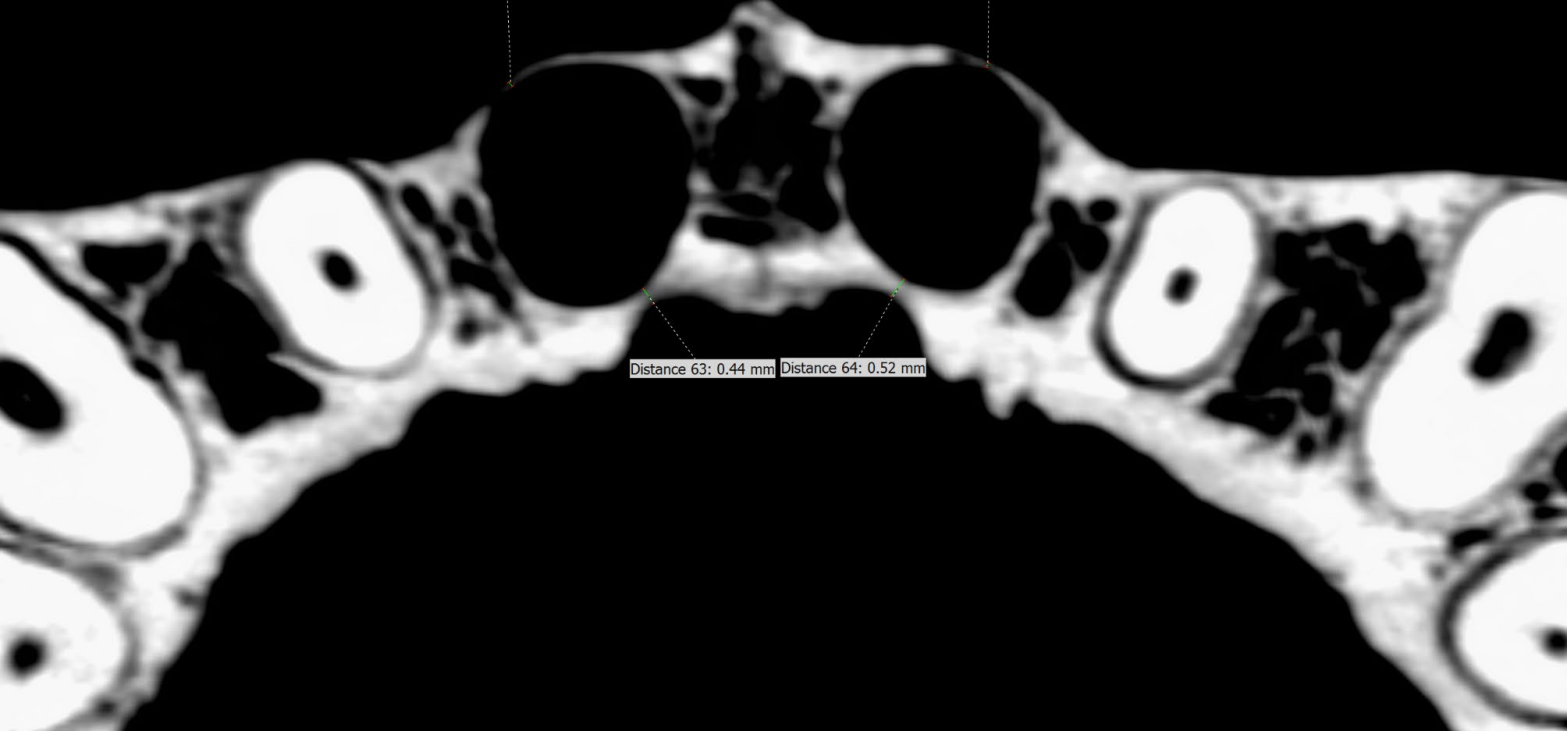
A measurement of the closest position of tooth #11/#21 on the palatal aspect to the ICO (Occlusial view)

The ICO’s distances in the axial plane to the roots of teeth #11 and #21 were measured. The slices were assessed in an apical to coronal direction. The measurement was made at the point at where the ICO’s diameter was at its largest, which was usually 0–3 mm below the crestal bone of tooth #11 or #21. The measurement was specifically collected from the location on the palatal aspect of tooth #11 or tooth #21 that was closest to the ICO (see Fig. 2).

### Reliability of measurements

Reliability of the measurements was performed by re-evaluating randomly 10% of samples twice after one-week interval and without any knowledge of previous measurements. In cases where discrepancies arose, a third evaluator was involved until adequate calibration was achieved. Intra-class correlation coefficients of 0.97 were achieved showing reproducibility of the evaluation of the categorical data. A kappa figure of 0.92 was achieved when comparing the numerical data, again showing good reliability of results obtained.

### Sample size

The practical limitations and past research precedents were taken into consideration when determining the sample size for this study. One hundred and twenty-three (123) human cadaver skulls were scanned using extremely accurate micro-XCT imaging. Following the application of stringent inclusion criteria, 15 skulls were eliminated, leaving 108 skulls in the final sample. The quantity of acceptable skulls available for scanning and the restricted scan time available, determined the selection of this sample size. Furthermore, most cited studies have employed comparable sample sizes, which guarantees conformity with accepted research procedures. The high-resolution data from micro-CT, despite the small sample size, offers a solid foundation for further investigation and allows for in-depth anatomical understanding that can guide larger-scale studies.

#### Ethics approval

was obtained from the Human Research Ethics Committee of the Faculty of Health Sciences, University of Pretoria, South Africa (No. 111/2013) and the Helsinki Declaration was signed.

### Statistical analysis

Statistical Analysis System (SAS 9.4) was used to conduct the statistical analyses. Descriptive statistics were calculated on all the variables in the analysis according to the different population and gender classifications of interest in this study. In order to compare mean measurement differences between multiple groups the non-parametric alternative to an Analysis of Variance (ANOVA) was considered, namely the Kruskal-Wallis test, due to the non-normality of the data. The non-parametric alternative to pairwise comparisons between different groups, namely the Dwass-Steel-Critchlow-Fligner (DSCF) test was also used to determine between which groups the mean differences were detected.

## Results

The mean age of the population was 54.48 years (SD:18.53; SE:1.79; 95% CI: 50.92–58.03(*n* = 108)) with 58.33% (*n* = 63) being male and 41.67% (*n* = 45) being female; and 48.15% (*n* = 52) being white and 51.85% (*n* = 56) being black. Demographic data of the subjects included in the study are displayed in Table 1.

**Table 1:** Demographic data

Population and gender	*n*	Average age (in years)
Black male	37	46.95 (± 15.68)
Black female	19	39.42 (± 15.66)
White male	26	63.35 (± 15.28)
White female	26	66.69 (± 14.51)
Total	**108**	**54.10 (± 18.53)**

The mean diameter of the ICO was 6.61 mm, while the ICL varied from 4.96 to 20.10 mm. The mean distances from teeth #11 and #21 to the ICO, were 1.88 and 1.83 mm, respectively. Table 2 shows the detailed descriptive analysis of all measurements performed across population groups and gender.

**Table 2:**
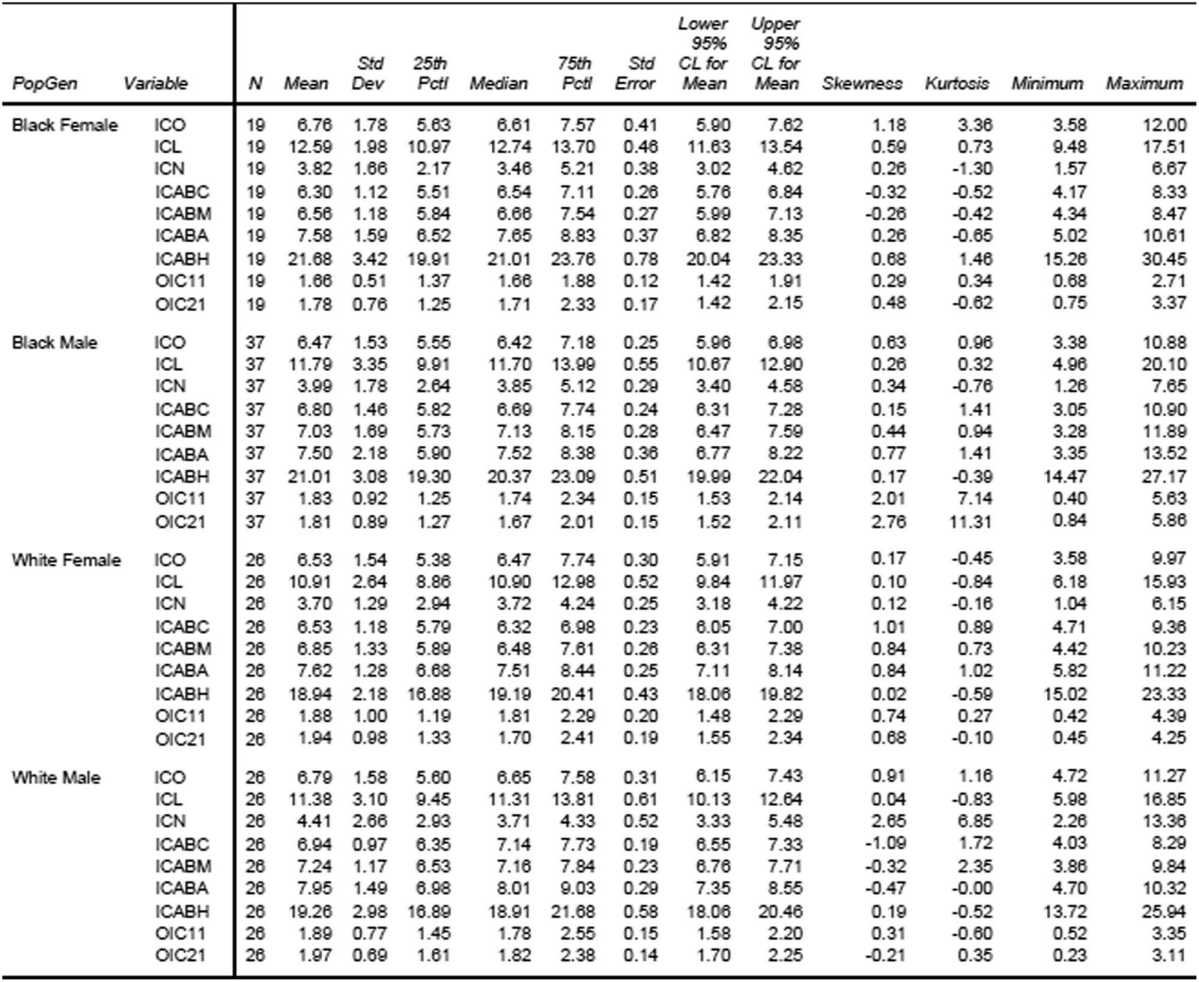
Descriptive statistics based on measurements across population groups and gender

Variables between population groups and genders for palatal bone measurements of OIC11 and OIC21 did not demonstrate significant differences. An interesting finding was that in 12.96% and 12.04% of cases for OIC11 and OIC21 respectively, the palatal bone was 1 mm or less from the maxillary IC.

**Fig. 3:**
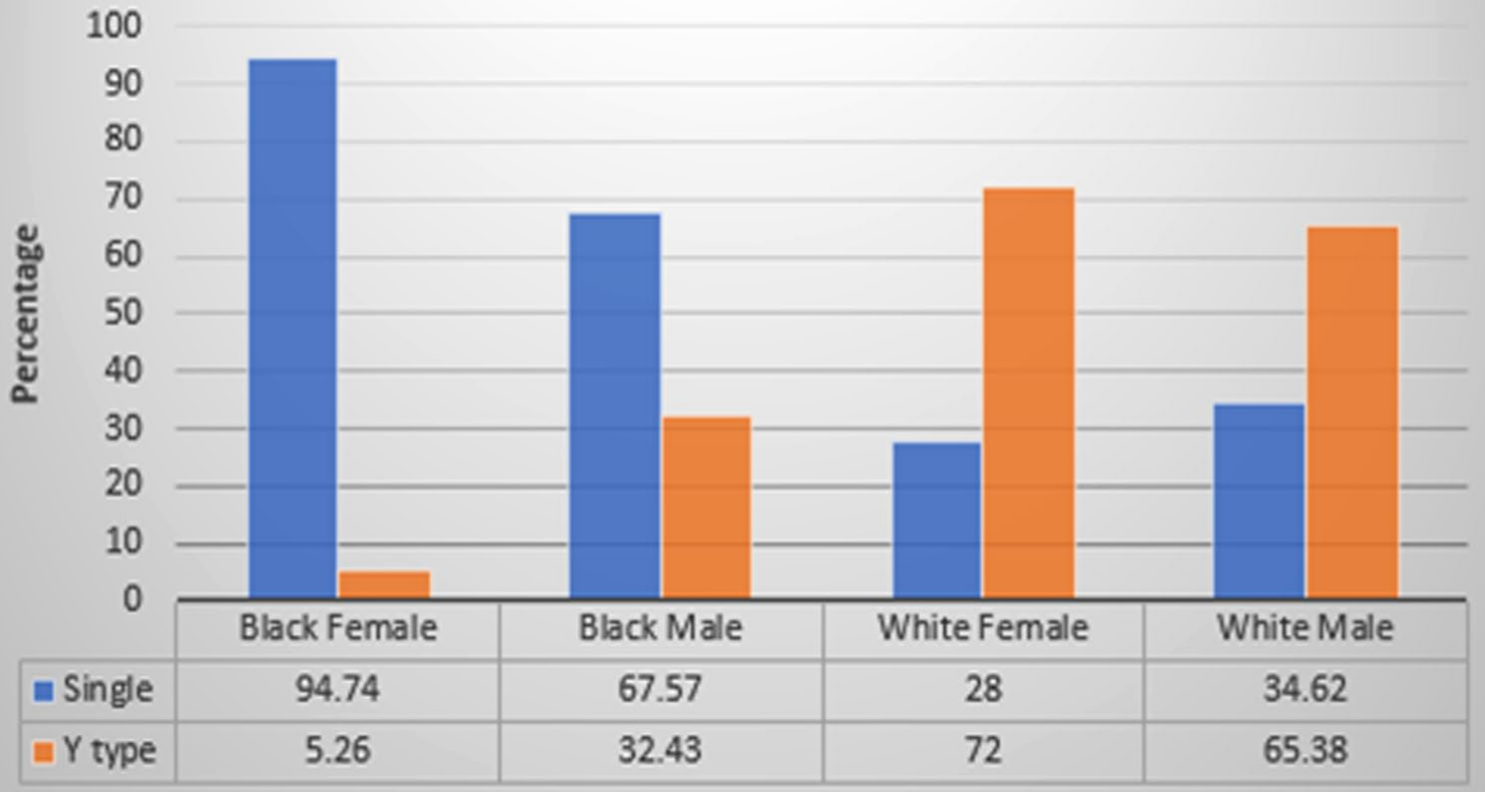
Distribution of different IC types between population groups by gender in coronal view *Note*: One category in coronal view (2-parallel canals) was excluded from this part of the analysis since only one skull (white female) exhibited this characteristic (*n* = 108)

When considering a possible relationship between population group by gender and different coronal views, namely Single canal and Y Type canal, there is very strong statistical evidence (Chi-square = 26.226, df = 3, *p* < 0.0001) to suggest that the coronal view is dependent on specific population and gender groups. Based on the coronal view a single canal being more prevalent in the black population and a Y-shaped canal being more prevalent in the white population (see Fig. 3).

In Fig. 3 it can be seen that 94.74% of the black female skulls (18 out of the 19 skulls) and 67.57% of the black male skulls (25 out of 37 skulls) had a single canal from the frontal view, whereas 72% of white female skulls (18 out of 25 skulls) and 65.38% of white male skulls (17 out of 26 skulls) had a Y type canal from the frontal view.

**Fig. 4:**
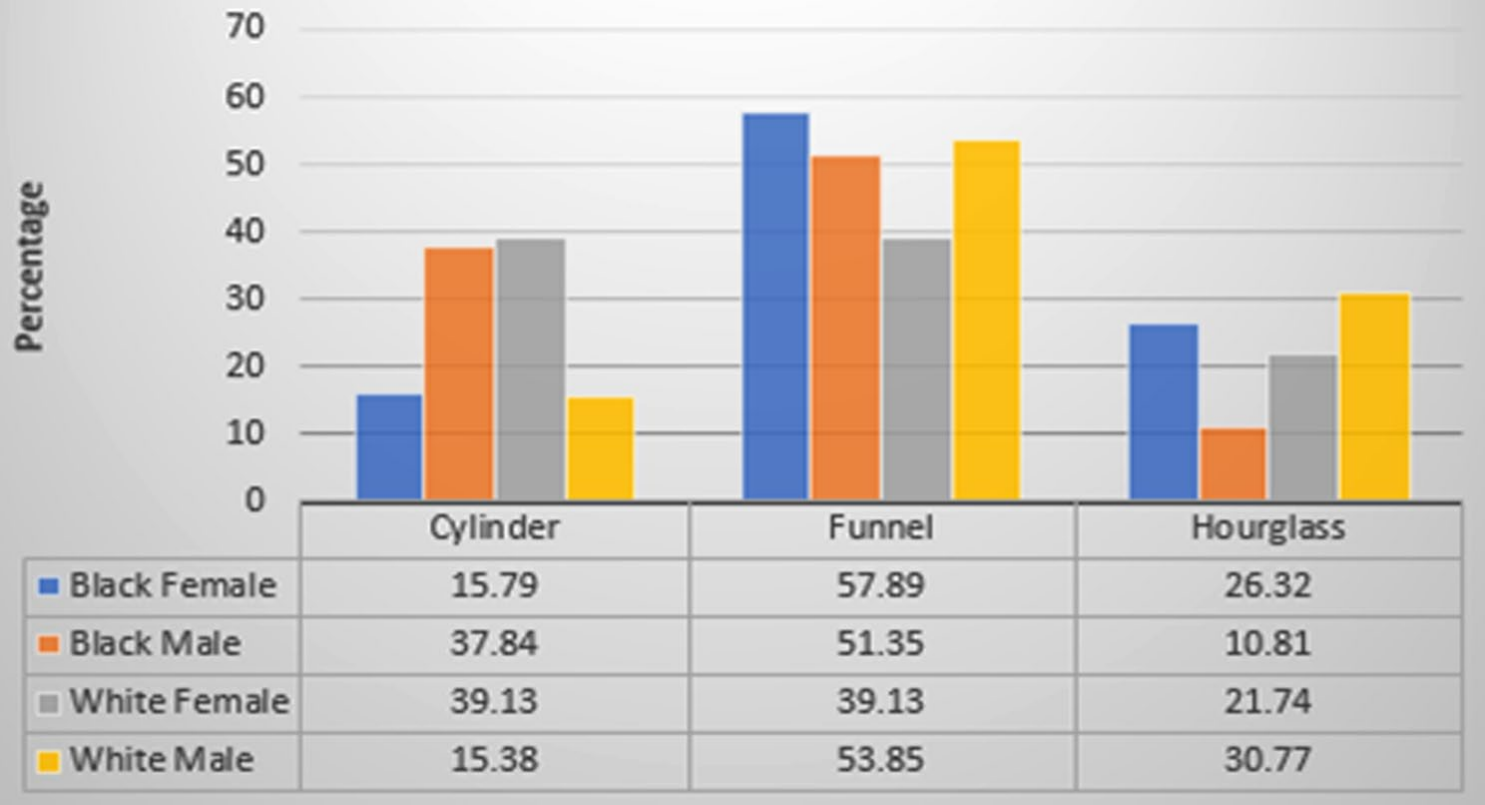
Distribution of different IC types between population groups by gender in sagittal view. *Note*: One category in sagittal view (Banana shaped) were excluded from this part of the analysis since only three skulls (white female) exhibited this characteristic (*n* = 105)

When considering a possible relationship between population group by gender and different sagittal views, namely cylinder, funnel and hourglass, there is no significance (Chi-square = 8.816, df = 6, *p* = 0.1842), suggesting that the sagittal view is not dependent on gender or population group (see Fig. 4).

When considering the ICABH and considering whether there are significant differences in this measurement across population groups and gender, it was found there is sufficient statistical evidence to suggest that there are differences in these measurements (Chi-square = 12.8228, df = 3, *p* = 0.005). Differences in ICABH measurements were found between black females and white females (*p* = 0.0134) and also between black males and white females (*p* = 0.0414). These results are presented in Table 3.

**Table 3:** ICABH measurements comparison within population groups and gender

Population/Gender	Wilcoxon Z	DSCF Value	Pr> DSCF
Black Female vs. Black Male	0.7182	1.0157	0.8899
Black Female vs. White Female	3.0220	4.2737	0.0134*
Black Female vs. White Male	2.2061	3.1199	0.1215
Black Male vs. White Female	2.6387	3.7316	0.0414*
Black Male vs. White Male	2.0733	2.9321	0.1618
White Female vs. White Male	-0.3752	0.5306	0.9820

The Dwass, Steel, Critchlow-Fligner method for pairwise comparison

p-value < 0.05 denoted by *

## Discussion

This study provides a thorough investigation of the anatomical features of the MIC in a South African population using micro-XCT. The high-precision micro-XCT is a superior imaging modality in comparison to conventional medical cone-beam computed tomography (CBCT) [38]. Both imaging modalities are based on the same principle, except the spatial resolution obtained is shifted to 1–3 microns (µm) with micro-XCT instead of 300 μm with CBCT. To obtain a high quality three-dimensional (3-D) virtual image at this high spatial resolution, the number of 2-D projections (radiographs taken in 360° of the sample) increases from 375 to up to 8 000 projections (1 000 projections for this study).

The cohort, which had 108 skulls with a mean age of 54.48 years, was divided into four racial and gender groups: 36 black males, 19 black females, 26 white males, and 26 white females. The breakdown of the population by age showed that the majority of black women were under thirty, black men were between forty and fifty, white women were over sixty, and white men were over seventy. Age-related variations in canal diameters are important because they affect how dental implants are placed and how other surgical procedures are carried out in the anterior maxilla. In our study, for example, younger people, especially those under thirty, typically have shorter canals. According to Bains (2023), age has a substantial impact on the MIC diameter, with mean values often rising with age [39]. This may have an impact on the size of implant used and the requirement for further grafting surgeries. Studies by Bornstein [9], Fernandez-Alonso [4], and Salemi F [15] have demonstrated that the success of dental implants and other surgical procedures may be impacted by age-related morphological changes in the MIC. These findings emphasize how crucial it is to take age-specific anatomical variations into account in order to maximize surgical success and reduce risks. For example, Bornstein [9] showed that diminished bone quality and greater bone resorption in older individuals increase the likelihood of implant failure, highlighting the necessity for customized surgical techniques. Age-related differences in canal dimensions support age-specific therapeutic considerations, as shown by our findings about gender variations in canal dimensions are consistent with those of Guncu [21] and Fernandez-Alonso [4], underscoring the need for surgical techniques tailored to each patient’s gender.

The analysis showed that the ICL had an overall span of 4.96 to 20.10 mm and a mean of 11.62 mm. These measurements highlight the substantial variation in ICL and are consistent with earlier research by Song [11], Bornstein [9], Guncu [21], Tozum [10], Fukuda [20], and Salemi [15]. However, significant gender-based variations in ICL, as proposed by Gonul [7], Fernandez-Alonso [4, 5], Bains [39] and Panda [8], were not supported by our results. Our findings showed ICO mean diameter to be 6.61 mm (range 3.38–12.00 mm). This value is slightly bigger than that reported by Mraiwa [6], Bornstein [9], Fukuda [20], and Salemi [15], and it is consistent with observations made by Gonul [7]. In the present study, an average ICN diameter was 3.90 mm (range 1.04–12.15 mm), which is less than that reported by Mraiwa [6] and Al-Amery [12], but similar to that of Bornstein [9] and Panda [8].

Our study found that the thickness of the alveolar bone anterior to the canal varied from 6.69 mm at the most coronal region to 7.65 mm at the most apical area, in accordance with the findings of Mraiwa [6], Bornstein (2011) [9], Tozum [10], and Al-Amery [12]. In particular, Mraiwa found a range of 2.9–13.6 mm for bone thickness, with a mean value of 7.4 mm [6]. The alveolar bone thickness measured by Bornstein was 7.6 mm at the apical part, 6.59 mm at the midsection, and 6.5 mm at the crestal area [9]. Tozum measured the thickness of the bone at the crestal portion (5.62 mm), the midsection (6.68 mm), and the most apical portion (9.19 mm) [10]. Al-Amery found the nasal spine’s thickest alveolar bone measured 10.75 mm, while the labial alveolus has the narrowest, measuring 5.7 mm, with an average thickness of 7.63 mm [12]. Furthermore, it was observed that the alveolar bone was often thicker in males [9, 10, 12]. It was also confirmed by Al-Amery [12] and Panda [8] that the thickness was higher in younger individuals. The mean height of the buccal alveolar bone was 20.21 mm (range 13.72–30.45 mm), which is significantly higher than Fukuda’s [20] findings. There were also significant disparities between black and white females, as well as black males and females.

The axial view distances from teeth #11 and #21 to the ICO, which were not disclosed in previous studies, revealed mean distances of 1.88 and 1.83 mm, respectively, indicating the crucial proximity of tooth sockets to the IC for anterior zone implant procedures. This is especially important when considering immediate implant placement to replace central incisors. In these cases, the drill is oriented more toward palatal to achieve sufficient primary stability and to stay away from engaging the buccal bone wall and may interfere with content of MIC. Our findings revealed that #21 and #11 tooth sockets are located in close proximity to important anatomical structures and highlighted the necessity for clinicians to use sophisticated imaging methods and perhaps guided implant placement procedures to reduce the risk of complications.

Our study identified clear associations between demographic factors and the MIC morphological features, especially regarding shape and prevalence in various coronal and sagittal perspectives. The analysis of coronal views showed the single canal shape to be more prevalent in the black population, while the white individuals were more likely to exhibit the Y-type canal. This finding is consistent with the studies by Gonul et al. [7] and Salemi et al. [15], which found that single-type canals were more common than Y-type canals. On the other hand, Fernandez Alonso et al. [3] found that Y-type canals were more common. Analysis in the sagittal plane revealed that the funnel form, which is found in all analysed demographic groups, is the most frequently encountered morphology of the MIC, followed by the cylindrical type. This distribution is consistent with findings published by Arnaut [40] and Fukuda et al. [20]. It does, however differ from the results of studies conducted by Tozum et al. [10], Guncu [21], and Gonul et al. [7], which reported a higher prevalence of cylindrical canal form. Because the MIC morphology varies so much, customized surgical planning is vitally important. This is particularly relevant to implant therapy in the anterior maxilla, where each patient’s unique anatomy must be considered when deciding whether to pursue MIC augmentation.

Our research offers novel information on the structural differences of the MIC in a South African community in addition to validating earlier findings. These results highlight the importance of tailored surgical planning that considers each patient’s unique anatomical differences in order to guarantee the best possible results for oral surgical treatments. Expanding the understanding of these anatomical traits in a variety of population groups should be the main goal of future research in order to improve clinical practice in oral surgery and implant dentistry.

### Study limitations

The study has several limitations, including a relatively small sample size of 108 human cadaver skulls, which restricts the application of the findings, suggesting the need for larger, more diverse studies. The focus on only two South African demographic groups further limits the applicability to other ethnic or racial populations. We acknowledge that age is a potential confounding factor in our study, given the nearly 20-year age difference between black and white groups. While we observed anatomical variations may be influenced by ethnicity, we recognize that some of these differences might also be attributed to the age disparity. Correlation but not causation may be established with the retrospective cross-sectional design, and a prospective longitudinal approach could offer deeper insights. Although micro-XCT scanning provides high-resolution pictures, it is resource-intensive it may not fully replicate in vivo conditions, highlighting the need for complementary imaging methods like CBCT. Clinically significant anatomical changes may have been overlooked due to the omission of skulls with specific diseases. Therefore, in order to enhance comprehension and therapeutic relevance, additional research involving bigger and more varied sample sizes is required, particularly in complex cases to provide stronger guidance for dental practitioners.

## Conclusion

This work offers a thorough analysis of the structural features and dimensions of the MIC using micro-XCT analysis of a South African cohort, revealing notable differences between demographic groups and genders. The study emphasizes that there are notable differences in the ICL and angulation, with males typically having longer canals than females. Additionally, it clarifies the intricate structure of the MIC by highlighting how near the central incisor sockets (#11 and #21) are—an average distance of less than 2 mm was found. These results imply that some demographic groups have a higher risk of surgical problems after dental implant surgeries. It emphasizes the significance of thorough preoperative evaluations using cutting-edge 3-D imaging technologies as it is the first study to our knowledge to take such detailed measurements from these tooth sockets. In order to minimize potential issues and improve the success of dental implants, it is imperative to customize surgical techniques based on the patient’s unique anatomical traits. This is particularly important when considering immediate implant placements in the demanding anterior maxillary region.

## Data Availability

The data that support the findings of this study are not openly available due to reasons of sensitivity and are available from the corresponding author upon reasonable request.

## Abbreviations

IC: Incisive canal
Micro-XCT: Micro-focus X-ray Computed Tomography
ICN Measurement: The diameter of the foramina of Stenson
ICO Measurement: The diameter of the incisive foramen
ICL Measurement: The incisive canal length defined as the distance from the incisive foramen to the foramina of Stenson
ICABC Measurement: The distance from the buccal border of the incisive foramen to the facial aspect of the buccal bone plate
ICABM Measurement: The measurement taken at the level opposite the palatal border of the incisive foramen to the facial buccal bone wall
ICABA Measurement: The distance from the buccal border in the middle of the incisive canal to the facial aspect of The buccal bone wall
ICABH measurement: The height of the buccal alveolar bone
OIC11 Measurement: The distance from the incisive foramen to the tooth #11
OIC21 Measurement: The distance from the incisive foramen to the tooth #21
CEJ: Cementoenamel junction
